# Comparative Study of the Gastric Mucosa of Risso’s Dolphin (*Grampus griseus*) and Bottlenose Dolphin (*Tursiops truncatus*): A Key to Manage the Diet in Captive Conditions

**DOI:** 10.3390/vetsci9100571

**Published:** 2022-10-16

**Authors:** Barbara Biancani, Livio Galosi, Adolfo Maria Tambella, Sara Berardi, Lucia Biagini, Subeide Mari, Giacomo Rossi

**Affiliations:** School of Biosciences and Veterinary Medicine, University of Camerino, 62032 Camerino, Italy

**Keywords:** diet management, dolphin, *Tursiops truncatus*, *Grampus griseus*, stomach histology

## Abstract

**Simple Summary:**

The bottlenose dolphin (*Tursiops truncatus*) is the most represented species housed in zoological settings in Western countries, while Risso’s dolphin (*Grampus griseus*) is usually kept under human care as a result of the rehabilitation process in rescue centers. Although they belong to the same family, Delphinidae, these two species present remarkably different feeding habits. Risso’s dolphin is strictly teutophagous, and its diet is particularly based on cephalopods such as mesopelagic squid, which is poor in histamine. The bottlenose dolphin is mainly ichthyophagous and primarily prefers blue fish, which is rich in histamine as a degradation product and stimulates the parietal cells of the stomach to secrete hydrochloric acid (HCl). These different eating habits are usually correlated to habitat, behavior and physiological needs related to the specific morpho-functional conditions of the species. Despite these dietary differences, in captivity, the two species are fed in the same way through the administration of several kilograms of bony fish (such as capelin or herring) per day. While this diet appears to be optimal for the bottlenose dolphin, Risso’s dolphin appears to be more sensitive, presenting gastrointestinal discomfort when ingesting bony fish for a few consecutive days.

**Abstract:**

To histologically evaluate the gastric compartments of Risso’s (*Grampus griseus*) and bottlenose dolphins (*Tursiops truncatus*) and provide suggestions for the diet of Risso’s dolphins in captivity, we examined 12 stomachs from both species. While slight differences in keratinization were observed in the forestomach, significant differences came to light in the second stomach’s mucosa. At this level, in Risso’s dolphin, the principal cells are markedly reduced in size and located externally to the parietal cells, not interspersed between them, compared to bottlenose dolphins; differences were also observed in the structure and concentration of the parietal and principal cells of the gastric body glands (*p* < 0.0001). The quantitative results of G- and D-cell counts in the gastric mucosa show a clear difference, with a higher concentration of G cells in the mucosa of Risso’s dolphin (t = 7.334; *p* < 0.0001) and a higher level of D cells in bottlenose dolphin mucosa (t = 3.123; *p* = 0.0049). These results suggest that parietal cells undergo greater stimulation by gastrin produced by G cells, with greater acid secretion in *G. griseus*. Further studies are needed to understand whether an inappropriate diet could lead to severe clinical signs due to gastric acidity in Risso’s dolphin.

## 1. Introduction

The gross anatomy of the digestive system has been studied in several species of cetaceans since the late 19th century [1,2,3,4,5], and the microscopic anatomy of different regions has been investigated in different species of toothed whales [6,7]. Although dolphins are polygastric animals, as they possess more than one stomach, the general physiological functions are comparable to the physiology of monogastric mammals. A complete review of the anatomy and physiology of the gastric compartment is beyond the scope of this paper, and extensive information is reported in the literature [7,8,9,10]. In cetaceans, the respiratory system and the digestive system are anatomically separated [11]. The esophagus opens into a saccular forestomach, and depending on the species, the gastric compartment consists of three or more chambers. In most dolphin species, there are three stomachs: the first stomach (forestomach, non-glandular) is mainly an enlargement of the esophagus, and it has a muscular and very distensible wall lined by a thick layer of stratified squamous keratinized epithelium lacking in cell nuclei, with irregular superficial dendritic cells that intensely stain with hematoxylin–eosin [6,12]. The forestomach is essential for grinding, squeezing and compressing unchewed food [11]. Gaskin [13] suggested that a certain amount of gastric juices, with a pH between 2.7 and 4.5 and refluxing into the pre-stomach from the second chamber, would help mechanical digestion by starting minimal enzymatic digestion. The first stomach does not have a rapid cell turnover, and it has no mucus-secreting glands [12]. The second stomach (fundic stomach, glandular compartment) presents a mucosa similar to that of monogastric mammals. It has a very limited capacity to expand, and the deep folds do not serve to increase elasticity [13] but to increase the surface area [12]. In the mucosa, there are gastric glands that contain neck cells, parietal cells and principal cells. The epithelium consists of mucoid cells that are responsible for the protection of the gastric mucosa from the harmful action of proteolytic enzymes and HCl by producing a mucous layer. Gastric glands consist of four types of cells: principal cells (approximately 7 µm), which produce digestive enzymes such as pepsinogen and lipase; parietal cells (approx. 15 µm), which secrete HCl in order to maintain the gastric pH at values between 2 and 4 and produce intrinsic factor, a glycoprotein essential for the absorption of cobalamin (vitamin B12); mucous neck cells (approx. 20–40 µm), which produce mucin and are present in abundance in the glands of *Phocoena* spp. and *Platinist* spp, while they appear absent or undifferentiable in *Pontoporia* spp., *Delphinus* spp., *Tursiops* spp. and *Stenella* spp. [13]; and neuroendocrine cells, distributed throughout the gastric mucosa and located mainly at the base and in the body of the glands. These cells have very electron-dense secretory vesicles near the nucleus, and, according to the content of the vesicles, the neuroendocrine cells of the stomach are divided into: G cells (producing gastrin that stimulates enterochromaffin-like cells to produce histamine and parietal cells to secrete HCl); D cells (producing somatostatin); enterochromaffin-like (ECL) cells; and enterochromaffin (EC) or argentaffin cells (producing serotonin). Cozzi et al. [12] reported the absence of argentaffin cells in the fundic stomach of dolphins. Neuroendocrine cells are also responsible for the secretion of factors that control gastric motility and glandular secretion. The fundic stomach communicates with the third stomach (pyloric stomach) through a narrow connecting channel possessing sphincteric constrictions. The pyloric stomach connects the gastric compartments, through a robust pyloric sphincter, to the duodenal ampulla of the small intestine, regulating the passage of digesta into the duodenum [14]. The third stomach’s mucosa has a columnar epithelium with mucous cells, gastric pits with tubular non-branching glands and argentaffin cells.

The morphology and histology of the different chambers have been interpreted in relation to feeding habits and digestion in different species of cetaceans, such as *Tursiops* spp., *Stenella* spp. and baleen whales [6,15,16,17], but currently, little is known about the gastric histology and physiology of Risso’s dolphin [18]. The bottlenose dolphin is the most represented species housed in a zoological setting, while Risso’s dolphin is sometimes kept under human care in rescue centers for the rehabilitation process. Although these are two species of the same family, Delphinidae, they present remarkably different feeding habits in the wild. Risso’s dolphin is strictly teutophagous, and its diet is particularly based on cephalopods such as mesopelagic squid [19,20,21], which is poor in histamine, while the bottlenose dolphin is mainly ichthyophagous [22,23] and primarily prefers oily fish, rich in histamine. These different eating habits are usually correlated to habitat, behavior and physiological needs related to specific morpho-functional conditions of the species.

The aim of the present study was to evaluate the histological differences between the gastric compartments of *Grampus griseus* and *Tursiops truncatus* and to provide indications for the diets of the two species in captivity through the characterization of the principal cells and the quantification of G and D cells in the glandular stomach (second stomach). 

## 2. Materials and Methods

### 2.1. Samples

Paraffin-fixed tissues from the stomachs of 12 bottlenose dolphins (*Tursiops truncatus*) were obtained from the Mediterranean Marine Mammal Tissue Bank of the University of Padova (Italy). From 12 Risso’s dolphins (*Grampus griseus*) stranded along the Italian coast over the years, fresh samples were collected during necropsy and fixed in 10% buffered formalin before being embedded in paraffin. No sample was collected from live animals, according to the Italian Legislative Decree 26/2014.

### 2.2. Morphological and Immunohistochemical Analysis

Paraffin-fixed samples of the stomachs were cut with a microtome (RM2035, Leica, Wetzlar, Germany), and 4 μm thick sections were set onto electrostatic slides (SuperFrost Ultra Plus©, Thermo scientific, Braunschweig, Germany) for maximum adhesion, allowed to dry and subsequently stained with hematoxylin–eosin (HE) for morphological evaluation. Slides were examined at different magnitudes under the microscope (DM2500, Leica, Wetzlar, Germany), at magnifications of 10, 20 and 40×. In five random fields, the number of principal cells in the glandular stomach was counted and scored from 0 to 3, and the intensity of cellular staining and the pattern of glandular tissue was also recorded and identified as pattern “a” or “b”, as described in Table 1. To evaluate the presence and number of G and D cells in the glandular stomach, an immunohistochemical analysis (IHC) was performed. Tissue sections were deparaffinized with successive xylene baths and then hydrated by passing through decreasing concentrations of alcohol baths and deionized water (100%, 95% and 70%) before proceeding to incubation with the following primary antibodies: Gastrin Polyclonal Antibody (PA5-16409, ThermoFisher Scientific, Walthman, MA, USA) and Somatostatin Polyclonal Antibody (PA5-16253, ThermoFisher Scientific, Walthman, MA, USA). Endogenous peroxidase activity was inhibited by using hydrogen peroxide (3%) in methanol for 20 min. Following peroxidase blocking, we proceeded to block nonspecific binding with 3% milk powder for 1 h in a 27 °C incubator and then rinsed the slides with TRIS solution. Later, sections were set in EDTA buffer at pH 9.0 and then in a microwave oven at 650 W for two cycles of 10 min to promote antigenicity. Samples were left at room temperature for at least 20 min and incubated with normal goat serum (Vector Laboratories Inc. corporate, Burlingame, CA, USA) before processing for primary antibody incubation. We detected antibody binding using ABC peroxidase (Vector Laboratories Inc., Burlingame, CA, USA) techniques with 1:200-diluted biotin-conjugated goat anti-rabbit immunoglobulin G (Vector Laboratories Incorporated, Burlingame, CA, USA), administered as a secondary antibody at room temperature for a 45-minute interval. The enzymatic reaction was revealed through the use of 3,1-diaminobenzydine (DAB, Sigma, St. Louis, MO, USA) as a substrate for the ABC-peroxidase procedure, with Meyer hematoxylin as a nuclear counterstain. The number of G or D cells was counted in 5 random fields. Histological and IHC assessments were performed blindly by a single operating pathologist. 

### 2.3. Statistical Analysis

Fisher’s exact test was used for the frequency analysis of principal cell organization patterns, and their scores were compared between groups with the Mann–Whitney test. The numbers of G and D cells were tested for a normal distribution using the Shapiro–Wilk test and analyzed with a nested t-test to compare the two groups of dolphins. Differences with *p*-values < 0.05 were considered statistically significant. All data were analyzed using the software GraphPad Prism 8 for MacOS, version 8.2.1 (GraphPad Software Inc., San Diego, CA, USA).

## 3. Results

**Figure 1:**
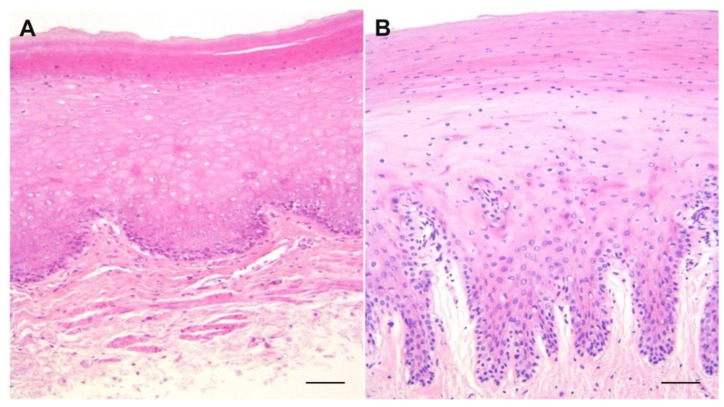
Histological sections belonging to bottlenose (**A**) and Risso’s dolphin (**B**) forestomach show similarity in the layers’ structures, with more pronounced and deeper development of chorion papillae in Risso’s dolphin than in bottlenose dolphin. Note that the keratinized layer is more pronounced in bottlenose than in Risso’s dolphin. H&E stain; bar = 250 µm.

The hematoxylin–eosin staining of samples of the forestomach (Figure 1) showed similarities in the layers’ structures, with more pronounced and deeper development of chorion papillae in Risso’s dolphin than in the bottlenose dolphin. The keratinized layer is characterized by anucleated and more compacted cells in the bottlenose dolphin, while Risso’s dolphin presents nucleated cells. 

**Figure 2:**
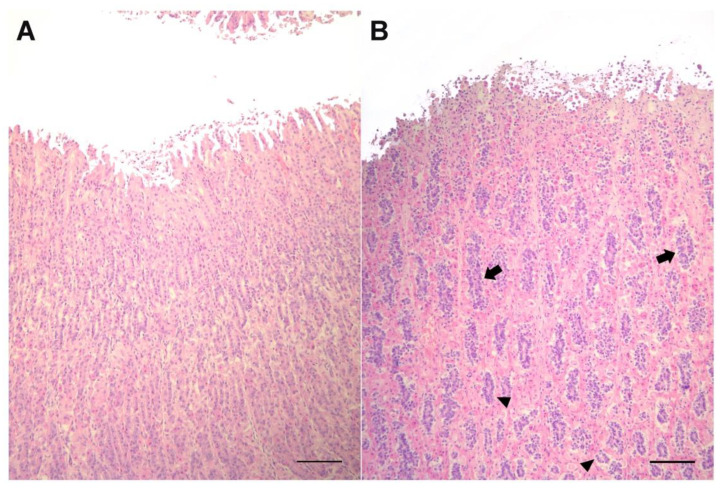
Histological sections of the second stomach from bottlenose (**A**) and Risso’s (**B**) dolphins. At this level, mucosa shows peculiarities in the number and pattern of arrangement of the principal cells in Risso’s dolphin, with a higher concentration of principal cells, smaller and strongly stained by hematoxylin. Note the arrangement of these cells in cords (arrows) more superficially or in an acinar pattern at deeper positions (arrowheads) in Risso’s dolphin. H&E, bar = 500 µm.

**Figure 3:**
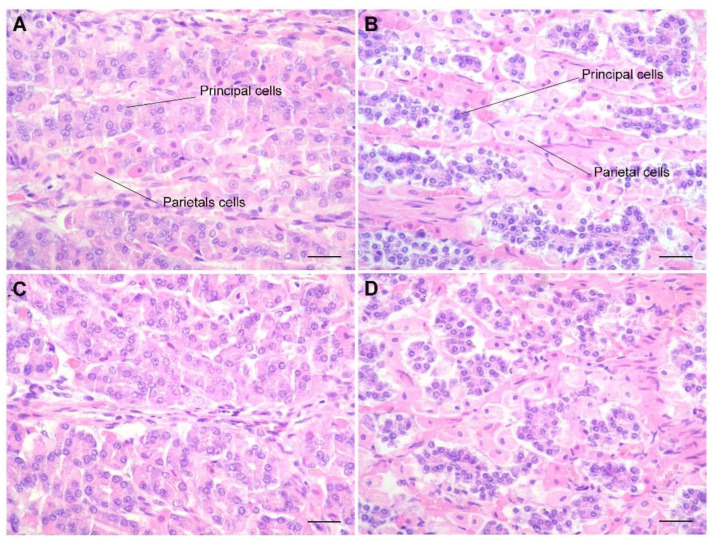
Bottlenose (**A**,**C**) and Risso‘s (**B**,**D**) mucosa of the second stomach. At higher magnifications, the mucosa presents evident morphological differences. In Risso’s dolphin, the principal cells are considerably smaller and strongly stained by hematoxylin compared to the main cells of bottlenose dolphins. They are also located outside the parietal cells, not intercalated between them. At this magnification, the greater concentration is even more evident, but also the cordoniform (**B**) and acinar disposition (**D**) assumed by the principal cells, strongly basophilic, in the mucosa from Risso’s dolphin compared to bottlenose (**A**,**C**). H&E stain; bar = 250 µm.

The mucosa of the second stomach (Figure 2) is comparable to the fundic portion of gastric mucosa in horses, dogs and pigs. In Risso’s dolphin, the principal cells are markedly reduced in size, although their number is greater, and strongly stained by hematoxylin compared to principal cells in bottlenose dolphins. They are located externally to parietal cells, not interspersed between them, thus giving the mucosa a laminar pattern that is more evident at a higher magnitude (Figure 3).

**Chart 1:**
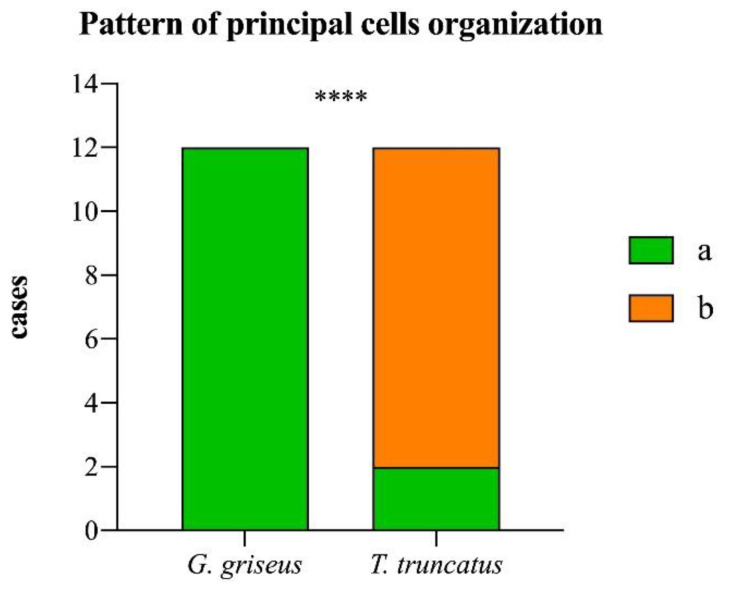
Frequency of cases showing principal cells organized in a “closed rosettes” pattern (**a**) or interspersed between parietal cells (**b**) in the two species of dolphins. Asterisks indicate a statistical difference between groups, ****: *p* < 0.0001.

In Risso’s dolphins, the mucosa has an acinar structure, but the differentiation between parietal and principal cells is still present, indicating that both mucous and pepsin are still produced at this level. The glands of the gastric body have a “double layer” tubular morphology: the internal layer, characterized by the presence of parietal cells, is characterized by strong eosinophilia and a more voluminous aspect; the external glandular layer, characterized by principal cells, is strongly marked by hematoxylin with intense blue color, tightened together to constitute a double layer that, in the coronal sections, has a cellular organizational aspect of “close rosettes”, while in the bottlenose dolphin, this pattern is less represented, and the principal cells are interspersed between parietal cells (*p* < 0.0001; Chart 1). 

**Chart 2:**
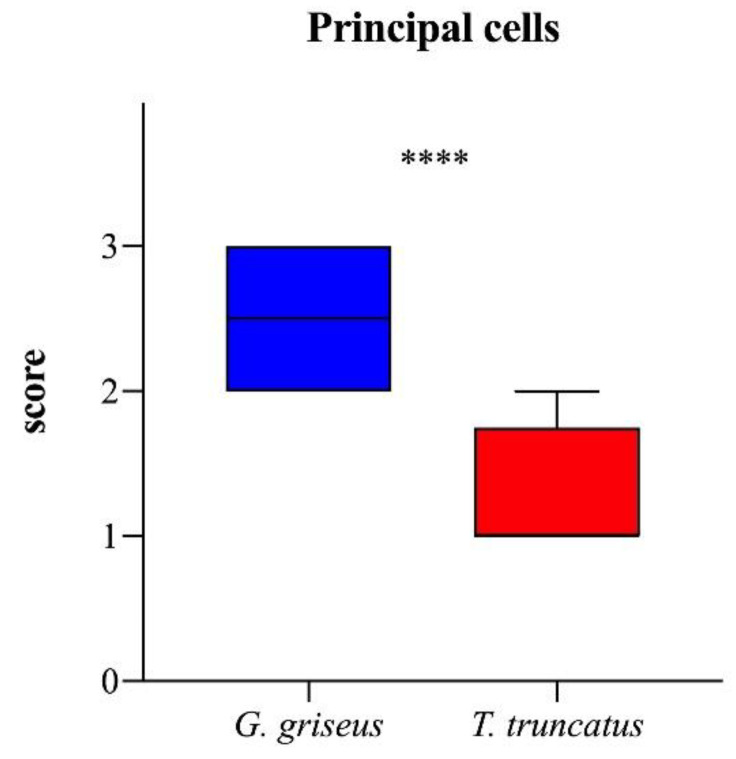
Boxplot showing the scores of principal cells in the two species of dolphins. The ends of the whiskers show minimum and maximum score values; boxes show the median and the first and third quartiles. Asterisks indicate a statistical difference between groups, ****: *p* < 0.0001.

This type of organization creates an almost virtual lumen within the glandular structures. Qualitative and semi-quantitative results for principal cells stained with HE showed a clear morphological difference between the two examined species. In the mucosa of the glandular stomach of Tursiops truncatus, the principal cells are much less present, scarcely stained by hematoxylin (resulting in a pale, clear, bluish color) and dispersed among the parietal cells (U = 9; *p* < 0.0001, Chart 2). In addition, the principal cells located mainly in the body and at the base of the gland are in a 3:1 ratio with the parietal cells (Figure 3).

**Figure 4:**
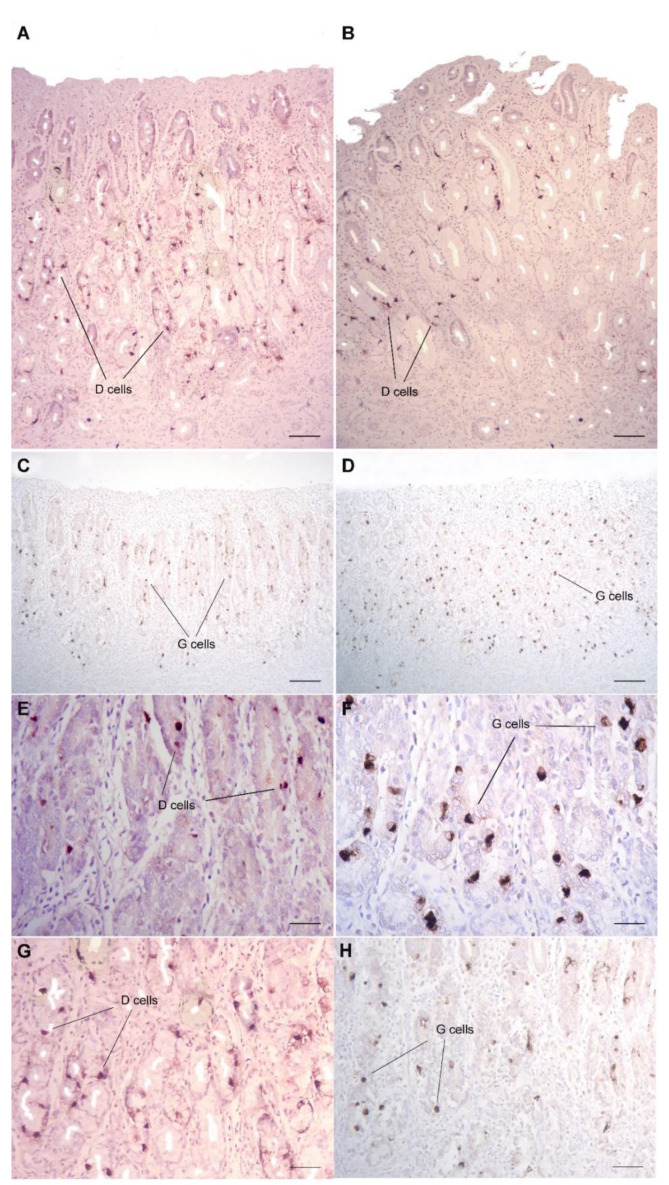
Immunostaining of G and D cells in bottlenose (**A**,**C**,**G**,**H**) and Risso’s gastric mucosa (**B**,**D**–**F**) of the second stomach. Although presenting a similar distribution pattern, Risso’s D cells were few and scattered in the mucosa (**B**,**E**), while its G cells were very abundant and diffusely expressed in the same mucosal areas (**D**,**F**). In contrast, in bottlenose dolphin, G and D cells were equally represented and alternated at the level of the mucosa (**A**,**C**,**G**,**H**). Anti-G-cell and anti-D-cell immunostaining, Harris’s hematoxylin counterstain, bar = 500µm (**A**,**B**), 600 µm (**C**,**D**) and 150 µm (**E**–**H**).

In Risso’s dolphin, this relationship of 3:1 is not respected, and the principal cells are present in greater numbers to create almost cords that entirely occupy the glandular wall (Figure 3). The quantitative results of G- and D-cell counts in the gastric mucosa of the bottlenose dolphin and Risso’s dolphin showed a clear difference between the two species, although presenting a similar distribution (Figure 4).

**Chart 3:**
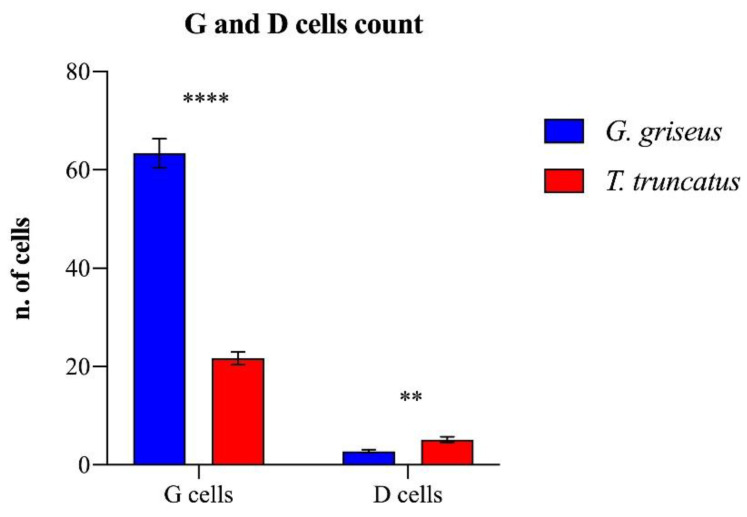
Mean number ± standard error of mean (SEM) of G and D cells in the glandular stomachs of the two species of dolphins. Asterisks indicate statistical difference between groups, ****: *p* < 0.0001; **: *p* < 0.01.

G cells are more abundant in the mucosa of Risso’s dolphin (t = 7.334; *p* < 0.0001). D cells, located in the glands near the pylorus, have a similar pattern in the two species, but the count was higher in the bottlenose (t = 3.123; *p* = 0.0049; Chart 3).

In the bottlenose dolphin, we observed an average of four D cells per gland, while in Risso’s dolphin, the number of these cells was considerably reduced, as only a single D cell per gland was present in most of the fields. The pyloric stomach presents glands organized in an acinar pattern. In the bottlenose, it has a typical antral mucosal structure producing mucous. Interestingly, the histological evaluation of the region between the fundic and pyloric stomachs highlighted the distribution of G cells, which were larger in size and present in significantly higher quantities in Grampus griseus than in Tursiops truncatus.

## 4. Discussion

The mucosa of the first stomach in the bottlenose dolphin shows a superficial parakeratotic layer and more mature underlying cells, with scarce cytoplasm and conspicuous desmosomes, which would predispose them to greater cellular cohesion and resistance [13]. These features of the mucosa guarantee the necessary resistance to mechanical trauma related to a diet with more compact and bony prey, as they are mainly ichthyophagous. Risso’s dolphin, on the contrary, has a diet based on softer and gelatinous cephalopods and presents a non-cornified epithelium with more immature cells. The fundic stomachs of the two species present histological differences regarding the density and distribution of glandular cells and the localization and density of parietal and principal cells. It has been proposed that, in cetaceans, the strong relationship between parietal cells and principal cells, in comparison with other mammals, could be correlated to the greater production of HCl necessary during the digestive process, since dolphins can ingest unpredictable quantities of food at irregular intervals, or to the low activity of protective cells [12] due to the presence of a smooth endoplasmic reticulum and underdeveloped intracellular canaliculi [6]. In Risso’s dolphin, the fundic gastric glands have a lower density of mucosecretory cells, leading to a less consistent and effective mucous barrier: less mucus and HCO3 secretion implies a consequent reduction in the barrier effect and the neutralization gradient. This condition would make the fundic mucosa of Risso’s dolphin more exposed to the HCl produced by parietal cells. A larger quantity of H+ ions could overcome the mucous barrier, and the reduction of HCO3- could be such that the buffering capacity of the mucus layer would not be guaranteed. This results in a pathological reduction in pH at the level of the apical surface of epithelial cells. The principal cells are dispersed among the parietal cells, similarly to that observed by Harrison et al. [6] in humans and in domestic mammals. The parietal cells produce digestive enzymes and are uniformly distributed up to the base of the gland. In the mucosa between the fundic and pyloric stomachs, we highlighted the distribution of G cells, which are larger in size and present in significantly higher quantities in *Grampus griseus* than in *Tursiops truncatus*. This suggests that the parietal cells undergo greater stimulation by the gastrin produced by G cells. D cells are responsible for somatostatin secretion, which has an inhibiting effect on gastrin production and on endocrine secretion from the pancreas. The lack of D cells and the reduction in somatostatin lead to a further increase in gastrin and thus a decrease in local pH. G cells regulate their activity mainly based on the state of filling of the stomach: when it is full, the walls are stretched, stimulating the production of gastrin, and the delomorphic cells are pushed to increase hydrochloric acid production by 6/8 times. Conversely, in an empty stomach, the walls are not stretched, the production of gastrin decreases (as hydrochloric acid also decreases), and the pH rises. The greater dilatability of the fundic stomach of *Grampus griseus*, compared to *Tursiops truncatus*, together with the greater concentration of G cells, would therefore lead to a greater stimulation of gastrin production. This, acting directly on enterochromaffin-like (ECL) cells, would potentiate the release of histamine, which would induce the parietal cells to activate gastric acid secretion. In normal conditions, hypergastrinemia is promptly corrected through classic feedback mechanisms. The principal inhibitory effect is granted by the HCl present in the stomach or duodenum, with a negative feedback mechanism: as soon as the gastric pH drops below 3, it causes the partial inhibition of the production of gastrin, and if a further reduction to 1.5 or less occurs, it stops the release of gastrin. HCl stimulates the D cells of the antrum to release somatostatin, thus inhibiting the secretion of gastrin and the release of histamine. However, if the density of D cells is lower, as it is in Risso’s dolphin, it is plausible to have a reduction in the feedback effect. Histamine, as a degradation product of the ingested fish, stimulates the parietal cells to secrete HCl. The bottlenose dolphin, due to the higher presence of D cells, is more resistant to histidine and gastrin and can easily have an intake of several kilograms of bony fish per day. In the authors’ experience, Risso’s dolphin appears to be more sensitive to the ingestion of bony fish, presenting gastrointestinal discomfort and constipation when ingesting 2–3 Kg of bony fish (capelin or herring) for a few consecutive days.

## 5. Conclusions

The present study shows that *Grampus griseus*, due to the lack of a keratinized layer in the mucosa of the first stomach, is particularly exposed to gastric secretions, and thus, its sensitivity to gastric acidity is more pronounced than in *Tursiops truncatus*. Although Risso’s dolphin is not one of the most typical species kept under human care, it is possible to have animals of this species in rescue centers for rehabilitation. Although further studies are needed to better understand what the appropriate diet is during the rehabilitation process of dolphins in captivity, our results suggest that within the *Delphinidae* family, some species may have different needs, and a diet based on bony fish could lead to gastric problems and/or a worsening clinical condition in *Grampus griseus*.

## Figures and Tables

**Table 1:** Methods to histologically evaluate the presence and pattern of principal cells in the glandular stomach. Evaluation performed at 40X High-Power Field (HPF).

	Score	Description
Presence of principal cells	0	None, 0–15 cells per HPF
	1	Scarce, 16–50 cells per HPF
	2	Moderate, 51–90 cells per HPF
	3	Abundant, >90 cells per HPF
Pattern of principal cell organization	a	Principal cells well stained with HE, organized in “closed rosettes”
	b	Principal cells poorly or scantly stained by HE and interspersed between parietal cells

## Data Availability

Data are contained within the current article.
